# Research methods in family medicine: an exploratory study of eleven years of congress programs using GPT-5

**DOI:** 10.1186/s12875-025-03145-w

**Published:** 2025-12-13

**Authors:** Jonas Cittadino, Jost Steinhäuser

**Affiliations:** Institute of Family Medicine, University Medical Center Schleswig-Holstein, Campus Lübeck, Lübeck, Germany

**Keywords:** General practice, Primary care, Comparative study, Data set, Large language models, German society of general practice and family medicine

## Abstract

**Background:**

Scientific conferences reflect trends in Family Medicine research and education. In Germany, the annual congress program of the German Society of General Practice and Family Medicine (DEGAM) encompasses a wide range of topics and is publicly accessible. However, little is known about how research methodologies and topics evolve over time.

**Methods:**

All program items from the DEGAM conferences from 2014 to 2024 were analyzed. Using the Large Language Model GPT-5, each item was automatically categorized by research methodology and topic. Descriptive statistics were used to summarize trends.

**Results:**

A total of 2,869 program items were identified. Quantitative and interventional studies constituted 53.5% of all methodologies, while qualitative and mixed-methods accounted for 33.1%. The relative proportions remained largely unchanged over the eleven-year period, however future-oriented topics such as eHealth or sustainability do emerge. Although diverse topics were represented, they showed no clear methodological evolution in every topic.

**Conclusions:**

This first exploratory analysis of a national Family Medicine conference series shows that, while the thematic range is broad, research methodologies remain stable. Therefore, as in patient care, academic Family Medicine is the ‘decathlon’ of health service research, too.

## Introduction

Family physicians (FPs) are expected to engage in lifelong learning to ensure the provision of up-to-date, evidence-based care. National and international societies support this goal by offering conferences that foster the exchange of scientific knowledge and clinical experience [1, 2]. Despite these efforts, the topics and research methodologies that shape the continuing education and academic discourse of Family Medicine remain largely influenced by cultural traditions and the organization of national healthcare systems [3, 4].

As academic general practice has expanded worldwide over the past decades [5, 6] and structured pathways for developing academic careers in Family Medicine have emerged [7, 8], understanding which research methods dominate the field and how they evolve might become increasingly important.

In Germany, the annual conference of the German Society of General Practice and Family Medicine (DEGAM) represents the central forum for academic exchange in the discipline [9]. Since 2014, the full conference programs, including abstracts and methodological details, have been publicly accessible online [10], showcasing all program items in form of an abstract, providing various information, including the research methodology of the study presented. As part of its core principles the society aims to “promote general medicine as a recognized scientific discipline” [11] and views the conference as a dialogue between FP and its academics. However, to date, no systematic analysis has explored how the scientific content and research methodologies presented at these conferences have developed over time.

The introduction of Large Language Models (LLMs), such as ChatGPT, has demonstrated an accurate understanding of various texts, including those with complex medical and scientific contexts [12–15]. This offers new opportunities for research in medical education and scientific communication, as demonstrated in recent studies using LLMs to summarize and classify medical abstract [16].

Thus, this study aimed to analyze trends in research methodologies and topics presented at DEGAM conferences over the past decade using GPT-5 and to examine their evolution over time.

## Methods

### Data

We aimed to capture a longitudinal overview of the methodological landscape represented in DEGAM conference programs. In the initial step, the websites of each year’s conference were identified, followed by a systematic transfer of the individual program items into a consolidated table. Due to the COVID-19 pandemic the annual conference in 2020 was combined with the annual WONCA Europe conference and held online [17]. Because the program had a more international focus and was structured differently, this year was excluded from the analysis [18].

### Extraction of specific topics

To get a quick overview of specific topics we defined four future-oriented topic and one Family Medicine-oriented topic. For each topic associated words or synonyms were defined and looked after in the program points. The following future-oriented topics were defined: One Health, sustainability, eHealth, and artificial intelligence. We included the following different writing styles and/or synonyms for each theme:

- One Health: “One Health”, “One-Health”, “OneHealth”.
- sustainability: “sustainabl*”, “sustainability”, “planetary health.
- eHealth: “e-health”, “ehealth”, “electronic health”, “digital health”, “m-health”, “mobile health”, “mobile-health”, “wearable”, “telemedicine”, “e-learning”, “elearning”, “digital health application”, “diga (German acronym for *digital health application*)”, “electronic prescription” (German: *E-Rezept*).
- artificial intelligence: “artificial intelligence”, “ai”, “machine learning”, “machine-learning”.

Further, we defined a family-medicine oriented topic according the European definition of general practice/Family Medicine and the Canadian Triple C Curriculum [19, 20] using the following words: “continuity”, “holistic”, “continuous”, “comprehensive” and “centered”.

### Extraction of methods

After an initial review of individual program items and in accordance with established research concepts of health services Research [21], the authors defined the following categories for methodological classification: cross-sectional study, cohort study, case-control study, qualitative study, mixed-methods, literature review, review article, intervention study, project presentation, planned study, workshop, case report, and unclear. We included project presentation and planned study for completeness of the conference record as they constitute a substantial and recurring part of the congress. To categorize each program item, we utilized OpenAI’s Automated Program Interface (API) and the “gpt-5” model. Initially, a seed for the reliability of the request was defined [22]. Using the following (translated in English) prompt, a methodology category for each program item was determined by the model: “Which of the following categories best fits the subsequent method description of a program item from the Congress of the German Society for General Medicine?” For all program points where the LLM could not assign a category the authors manually choose a corresponding category. For further analysis we grouped cross-sectional, cohort, and case-control studies under the term quantitative studies [21].

### Extraction of topics

For the classification of topics, we utilized those already identified regarding dissertation topics [23]: complementary medicine, screening measures, reasons for encounter, teaching, basic psychosomatic care, prescription medication, practice management, hypertension, diabetes, medicine of the elderly, out-of-hour care, palliative care, interdisciplinary cooperation, stroke, house visits, obesity, quality management, multimorbidity, back pain, diagnostics, unclear. For categorization, we also used OpenAI’s API and the “gpt-5” model with the following (translated) prompt: “Tell me, which of the following topic descriptions best fits the subsequent description of a program item from the Congress of the German Society for General Medicine?” After an initial review, analysis of “unclear,” and experience reports, we expanded these topics to include Covid-19, depression, Chronic Obstructive Pulmonary Disease (COPD), coronary heart disease (CHD), cancer, reasons for working in rural areas, reasons for establishing a practice, and reasons for choosing to specialize in general medicine.

### Statistical analysis

We performed descriptive analyses (absolute and relative frequencies) and compared temporal trends visually using bar and pie charts. No inferential statistics were applied due to the exploratory nature of the study. For all analyses we used Python version 3.11 [24]. Analyses using GPT-5 were performed using the following hyperparameters:

- Reasoning = “effort”:”minimal”,
- Max_output_tokens = 1000,
- Text = “format” : {“type” : “json_schema”}.

### Ethical approval

Due to the study design and the data being publicly available no ethical approval from the institutional review board was needed.

## Results

### Characteristics of the data

As the 2020 conference was excluded due to the combination with the annual WONCA Europe conference during the Covid-19 pandemic, program items could be extracted for ten out of eleven years. While there were fewer than 200 program items in the first two years, the number in the following years was significantly higher, reaching over 300 program items in the last three years. In total, 2,869 program items from nine years were analyzed (Table 1).

**Table 1:** Program items and future-oriented vs. Family-Medicine based topics

	One Health	sustainability	eHealth	artificial intelligence	Family Medicine	program items	frequency (%)
2014	0 (0.0)	2 (1.1)	0 (0.0)	0 (0.0)	16 (8.8)	182	6.34
2015	0 (0.0)	2 (1.1)	0 (0.0)	0 (0.0)	18 (10.3)	174	6.06
2016	0 (0.0)	9 (3.0)	3 (1.0)	1 (0.3)	31 (10.3)	300	10.46
2017	0 (0.0)	17 (5.2)	5 (1.5)	3 (0.9)	30 (9.1)	330	11.5
2018	0 (0.0)	9 (3.2)	8 (2.9)	0 (0.0)	17 (6.1)	277	9.65
2019	0 (0.0)	15 (5.3)	12 (4.2)	1 (0.4)	38 (13.3)	285	9.93
2021	0 (0.0)	14 (4.4)	18 (5.6)	5 (1.6)	43 (13.4)	320	11.15
2022	0 (0.0)	19 (6.1)	9 (2.9)	2 (0.6)	46 (14.8)	310	10.81
2023	0 (0.0)	25 (7.6)	12 (3.6)	3 (0.9)	58 (17.6)	329	11.47
2024	0 (0.0)	21 (5.8)	15 (4.1)	8 (2.2)	50 (13.8)	362	12.62
Total	0 (0.0)	133 (4.6)	82 (2.9)	23 (0.8)	347 (12.1)	2869	100

Table showing the distribution of future-oriented topics, the Family Medicine-based topic and all program items with their amount and respective frequencies for each year

Four future-oriented topics represented less than 5% each with *sustainability* being the most prominent topic. Their distribution fluctuated each year with no clear identifiable trend. *One Health* was never represented. On contrast, the *Family-Medicine topic* was more often present, accounting for a total of 12.1% of all program items

### Methods used

Overall, cross-sectional studies constituted the largest group, making up 23.6% (*n* = 678) of all program items (Table 2). Since 2016, there has increasingly been a variety of workshops, making them the second-largest group with 17.8% (*n* = 511) items. Case-control studies, case reports and planned studies were scarcely represented (Table 2). Of the program items 9% (*n* = 259) could not be assigned to a methodology category by the LLM and were manually assigned to the right corresponding category by the authors.

Despite annual variations in the number of program items, the relative proportions of quantitative, qualitative, and mixed-method approaches remained stable.

**Table 2:** Assigned methodology for each year

Used methodology	2014	2015	2016	2017	2018	2019	2021	2022	2023	2024	Total (%)
cross-sectional study	67	54	64	80	66	58	80	59	60	90	678 (23.6)
workshop	2	3	55	80	68	72	58	63	56	54	511 (17.8)
qualitative study	31	37	52	32	29	25	50	40	52	44	392 (13.7)
project presentation	12	12	24	45	31	37	30	30	40	47	308 (10.7)
intervention study	23	25	34	30	27	32	33	24	19	41	288 (10)
mixed-methods	17	12	21	12	17	21	31	31	31	39	232 (8.1)
literature review	5	12	14	26	14	11	4	23	25	15	149 (5.2)
cohort study	11	12	18	11	14	6	15	17	21	14	139 (4.8)
review article	12	6	12	8	7	15	9	14	17	12	112 (3.9)
planned study	1	1	4	4	4	6	6	8	7	4	45 (1.6)
case report	1	0	0	1	0	2	3	1	1	1	10 (0.4)
case-control study	0	0	2	1	0	0	1	0	0	1	5 (0.2)

Table showing the distribution of assigned used methodology category and all studied years

### Topics

All program items could be assigned to one of the predefined categories. Over the years, “teaching” was the most common theme, accounting for 22.1% (*n* = 629). Interdisciplinary cooperation 9.2% (*n* = 261), prescribing medication 8.6% (*n* = 246), reasons for encounter 7.1% (*n* = 203), and practice management 6.9% (*n* = 196) followed as the next most common themes. However, starting in 2021, Covid-19 consistently occupied a prominent part of the program (Table 3). Themes like stroke, obesity, COPD and cancer were among the least addressed, each with under 20 program items. Topic categories were diverse but showed no systematic methodological evolution.

**Table 3:** Assigned topics for each year

Topic	2014	2015	2016	2017	2018	2019	2021	2022	2023	2024	Total (%)
teaching	26	22	64	73	73	63	66	75	72	95	629 (22.1)
interdisciplinary cooperation	17	17	26	22	25	20	31	33	40	30	261 (9.2)
prescription medication	22	23	32	32	26	33	21	18	16	23	246 (8.6)
reasons for encounter	9	15	20	30	18	25	19	18	28	21	203 (7.1)
practice management	11	6	17	19	15	21	26	26	21	34	196 (6.9)
quality management	8	16	21	10	18	17	8	15	24	16	153 (5.4)
basic psychosomatic care	5	11	17	22	19	13	11	14	16	23	151 (5.3)
Covid-19	0	0	0	0	0	0	41	26	23	24	114 (4)
palliative care	9	8	3	15	10	11	19	9	11	8	103 (3.6)
medicine of the elderly	14	7	14	6	12	8	10	8	5	8	92 (3.2)
screening measures	9	5	7	7	6	6	8	6	10	14	78 (2.7)
depression	7	4	7	3	6	8	10	7	7	13	72 (2.5)
reasons for working in rural areas	5	4	9	3	5	7	6	11	10	9	69 (2.4)
reasons for choosing to specialize in general medicine	4	4	8	10	5	4	5	7	8	14	69 (2.4)
diabetes	3	6	9	7	2	4	8	4	8	2	53 (1.9)
multimorbidity	7	7	6	4	4	5	3	5	5	4	50 (1.8)
complementary medicine	4	2	10	11	3	6	4	1	2	3	46 (1.6)
coronary heart disease	2	1	7	5	4	5	2	5	5	3	39 (1.4)
unclear	0	0	0	30	2	7	0	0	0	0	39 (1.4)
out-of-hour care	2	1	6	4	4	2	4	4	2	4	33 (1.2)
reasons for establishing a practice	0	0	4	4	2	2	5	3	5	0	25 (0.9)
house visits	2	5	5	3	0	4	1	1	1	2	24 (0.8)
hypertension	6	2	3	1	2	1	5	2	0	1	23 (0.8)
back pain	2	3	1	1	4	0	4	5	1	1	22 (0.8)
cancer	1	1	3	2	3	1	0	2	3	3	19 (0.7)
COPD^1^	2	2	0	4	3	5	0	0	1	1	18 (0.6)
obesity	1	0	1	2	2	3	0	0	2	5	16 (0.6)
stroke	3	1	0	0	2	3	0	0	0	0	9 (0.3)

Table showing the distribution of assigned topic category and all studied years. ^1^COPD = Chronic Obstructive Pulmonary Disease

The distribution of categories remained constant over the years, although there were exceptions for some categories: the relative share of “teaching” increased over the years, and with the onset of the COVID-19 pandemic, this theme consistently represented a relevant portion of the program items.

**Fig. 1:**
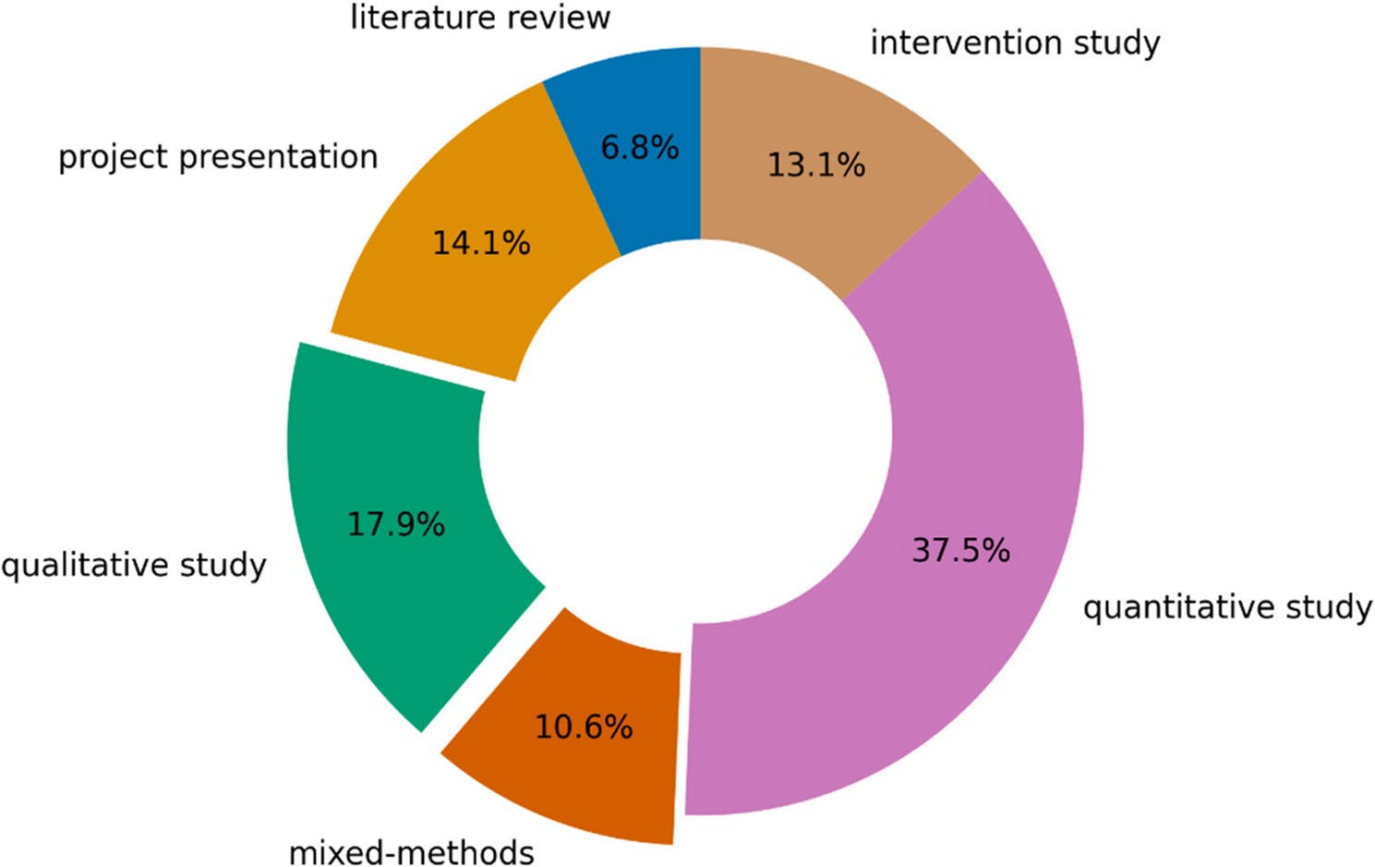
Used methodology for all program items. Pie chart depicting the distribution of the assigned methodology for all program items (n=2869) with highlighting the part which contain qualitative research methods (excluding the categories review article, project presentation, planned study, workshop, case report, and unclear). Quantitative research methods consist of cross-sectional, cohort and case-control studies

Of all program items 28.5% involved studies or projects with qualitative research methods or mixed methods, and 50.6% were attributable to quantitative and intervention studies (Fig. 1).

**Fig. 2:**
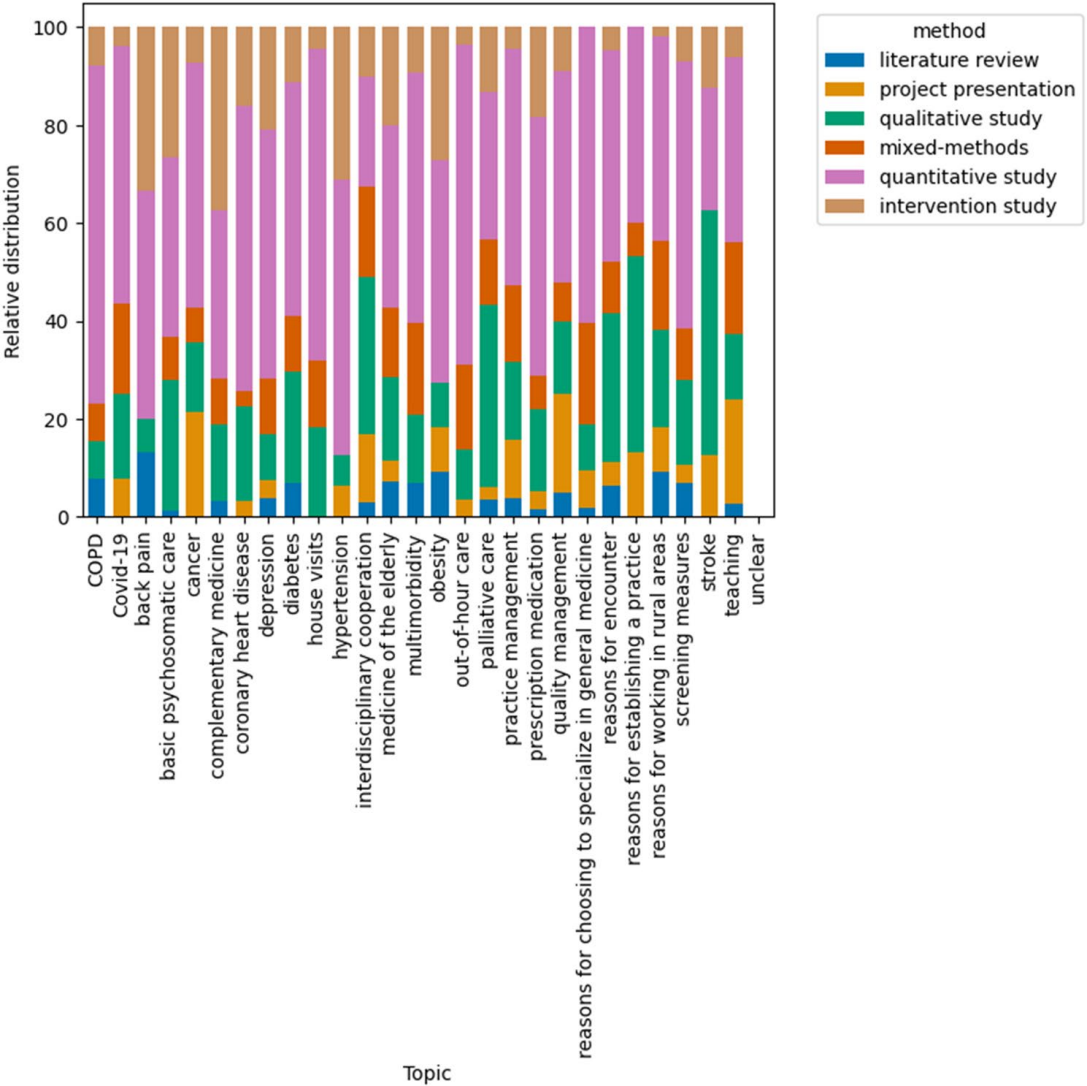
Used methodology for each topic. Bar chart showing the relative distribution of assigned methodology for each assigned topic for all years taken together (excluding the categories review article, project presentation, planned study, workshop, case report, and unclear). Quantitative research methods consist of cross-sectional, cohort and case-control studies

A few themes, such as “interdisciplinary cooperation”, “reasons for establishing a practice”, “palliative care“ and “reasons for working in rural areas” were characterized by a higher proportion of qualitative methods (Fig. 2).

## Discussion

### Main findings

This exploratory study provides the first longitudinal, GPT-5-assisted analysis of research methodologies and topics presented at national Family Medicine conferences. Across more than 2800 program items spanning eleven years, the methodological composition of research remained remarkably stable. While a wide spectrum of subjects was addressed, no topic showed clear methodological evolution within itself. The underling factors influencing this finding should be addressed in future analysis.

### What is already known

Recently, an analysis of dissertations within the archive of German-speaking Family Medicine (ADAM) provided a temporal representation of the wide range of general medical research topics [23]. This study demonstrated how the share of qualitative research methods has increased since the 1960s and constitutes about a fifth of the more recent dissertations [23]. In contrast, our findings suggest that the methodological profile of conference contributions has not changed substantially in the past decade. This discrepancy could indicate that innovative or early-phase methodological work is also disseminated through other academic channels. To what degree this finding might also be a matter of missing literature sources e.g. due to unpublished doctor thesis or limitations in literature reviews, should be a matter of future research.

Additionally, there might be an even stronger external influences on the evolution of Family Medicine as a recent national analysis of self-declared research priorities of German medical schools showed: A strong concentration on biomedical and laboratory-based disciplines was shown, whereas areas such as primary care and health services research remain underrepresented [25]. This undoubtedly has relevant influence on financing of Family Medicine research - as well as providing primary care.

Recent advances in LLMs have opened new possibilities for analyzing scientific communication. Prior work has already shown that models such as GPT-4 can accurately summarize and classify medical content [12–16]. A recently published analysis was able to demonstrate the utility of the LLM ChatGPT in the automated summarization of scientific articles, seeing its benefit in allowing general practitioners to quickly gain an overview of current research topics [16]. Our study builds on this foundation by demonstrating the feasibility of using GPT-5 to perform large-scale meta-research on academic conference data.

### Implications for research and education

The public availability of DEGAM conference programs enabled a comprehensive overview of research activities within German Family Medicine over more than a decade. The consistent dominance of quantitative and intervention-based methods may reflect a mature research tradition and a well-established methodological repertoire. Therefore, academic Family Medicine includes all methods of health services research giving physicians aiming at this career the opportunity of being holistic health services researchers, too.

The observed methodological stability raises the question of whether this represents scientific maturity or if barriers for further development have not yet been identified. While a consistent methodological core may reflect the consolidation of established research traditions, the limited visibility of approaches, such as pragmatic clinical trials, mixed-methods designs and participatory action research suggests room for methodological innovation in family medicine research.

Future-oriented topics such as sustainability, eHealth, and artificial intelligence were increasingly represented, but still accounted for a small share of program items, suggesting that the conference only gradually adapts to emerging societal developments. This pattern aligns with broader trends in German academic medicine, where research priorities remain predominantly biomedical, with comparatively limited focus on primary care and health-services research [25]. Understanding why certain themes, such as “reasons for working in rural areas” remain methodologically rather static may help identify structural barriers to methodological advancement.

Understanding how research methods evolve in Family Medicine is essential for shaping academic training and conference design. These findings might therefore especially offer orientation for early-career researchers and educators: they highlight which methodological competencies currently dominate academic discourse in Family Medicine, and where expansion might enrich the field. More broadly, this analysis demonstrates the potential of LLMs to assist meta-research and to inform academic planning in Family Medicine.

### Strengths and Limitations

To our knowledge, this is the first study to use an LLM to analyze an entire national conference program series in Family Medicine. The GPT-5 model enabled efficient categorization of a large corpus of abstracts, illustrating a novel approach to meta-research in primary care. Nevertheless, LLM-based classification is not without limitations [26, 27]: model bias and misclassification cannot be entirely excluded, even though random validation checks showed satisfactory accuracy.

A further limitation is that conference programs capture only a portion of German academic Family Medicine. Important research may also be disseminated through other outlets, such as publications, institutional reports, or international collaborations. Consequently, our findings reflect the visible conference discourse rather than the full scope of scholarly activity. In addition, our analysis was restricted to a single national context where comparative studies across other countries’ conferences could provide a broader understanding of methodological evolution in Family Medicine research. Therefore, future research should examine the reasons for this stability and include other sources of academic work to provide a broader understanding of methodological development of topics in Family Medicine. 

## Conclusions

This exploratory study shows that the methodological spectrum of Family Medicine research presented at DEGAM conferences is rich and has remained largely stable over the past decade. Although the thematic breadth is wide, not all topics exhibit a clear methodological evolution. These findings may help guide methodological training and support targeted thematic development for the field in future.

## Data Availability

Data will be made available from the corresponding author upon a reasonable request.

## Abbreviations

ADAM: Archiv der deutschsprachigen Allgemeinmedizin (English: archive of German-speaking Family Medicine)
API: Automated Program Interface
CHD: Coronary Heart Disease
COPD: Chronic Obstructive Pulmonary Disease
DEGAM: Deutsche Gesellschaft für Allgemeinmedizin und Familienmedizin (English: German Society of General Practice and Family Medicine)
EBM: Evidence-Based Medicine
FP: Family physician
GPT: Generative Pre-Trained
LLM: Large Language Model
USA: United States of America
WONCA: World Organization of Family Doctors
